# Supplementary material for: Object detection as an aid for locating the prostate in surface-based abdominal ultrasound images
Source: Commun Eng. 2025 Nov 28;4:217. doi: 10.1038/s44172-025-00550-y (PMC12728191; doi:10.1038/s44172-025-00550-y)
Supplement: Supplementary file 2 — Object Detection as an Aid for Locating the Prostate in Surface-Based Abdominal Ultrasound Images [file 44172_2025_550_MOESM2_ESM.pdf]

Object Detection as an Aid for Locating the Prostate in Surface-Based Abdominal Ultrasound Images – Supplementary Material

Rory Douglas Bennett[1], Tristan Barrett[2], Vincent J. Gnanapragasam[2], Zion Tsz Ho Tse[1*]

[1] School of Engineering and Materials Science, Queen Mary University of London, Mile End Road, London, UK, E1 4NS.

[2] Department of Radiology, University of Cambridge School of Clinical Medicine, Cambridge, UK, CB2 0QQ.

*z.tse@qmul.ac.uk

Supplementary Table 1: Training and testing hardware setups used for all models.

	**Python Version**	**PyTorch Version**	**GPU**
Training Hardware	3.10	2.5.1+cu124	HPC assigned Tesla and Ampere Nvidia GPUs.
Testing Hardware	3.12	2.5.1+cu124	Nvidia 4060 Laptop (6GB VRAM)

Supplementary Table 2: Parameter values used during training of the YOLO, RetinaNet, and FasterRCNN models. Any parameters not in this list had default values used. NA indicates a parameter that was not applied to a specific model.

**Parameter**	**YOLO**	**RetinaNet/FasterRCNN**
epochs	1000	1000
imgsz	600	600
pretrained	False	False
batch	8	32
cos_lr	True	NA
patience	100	100
val	True	True
shear	15	15
mosaic	0	NA
fliplr	0.2	0.2
scale	0.3	0.3
dropout	0.2	NA
hsv_h	0	NA
hsv_s	0	NA
hsv_v	0	NA
degrees	30	30
translate	0.2	0.2
erasing	0.5	Probability=0.5; scale=(0.02, 0.08)
Oversampling Factor	NA	8
Gradient Clipping	NA	True
Optimiser	NA	Stochastic Gradient Descent
Optimiser Learning Rate	NA	0.01
Optimiser Momentum	NA	0.9
Optimiser Weight Decay	NA	0.005
Learning Rate Schedular	NA	Cosine Annealing with Warm Restarts
Learning Rate Schedular Restart	NA	1000
Gaussian Noise	NA	mean=0.5; sigma=0.1
Gaussian Blur	NA	5x5 matrix

Supplementary Table 3: 5-fold cross-validation metric numeric results. All metrics were calculated for all detected prostate bounding boxes except IoU. Bold values indicate best performing model per metric. Confidence is given as the average confidence of the model across all patients for the highest confidence bounding box.*

**Model**	**IoU***	**IoU**	**Precision**	**F1**	**Recall**	**AP50**	**AP75**	**AP50-95**	**Confidence**
$YOLO^{P}$	0.74	0.7	0.92	0.92	0.92	0.89	**0.43**	**0.51**	0.62
$YOLO^{PB}$	0.72	0.67	0.88	0.88	0.88	0.85	0.43	0.46	0.7
$FasterRCNN^{P}$	0.74	0.67	0.87	0.87	0.87	0.82	0.4	0.44	0.93

FasterRCNN^{PB}	0.72	0.67	0.81	0.81	0.81	0.74	0.4	0.42	0.94
RetinaNet^{P}	0.73	**0.7**	**0.93**	**0.93**	**0.93**	**0.9**	0.42	0.47	0.88
RetinaNet^{PB}	**0.75**	0.68	0.82	0.82	0.82	0.74	0.4	0.41	0.93

Supplementary Table 4: 5-fold cross-validation PCC and RMSE numeric results. Only the highest confidence bounding box was used for the inferred dimension calculations. SI, RL, and AP RMSE values are in mm while the Volume RMSE value is in cm^3. Bold values indicate best performing model per measurement.

	**PCC**				**RMSE**			
**Model**	**SI**	**RL**	**AP**	**Volume**	**SI**	**RL**	**AP**	**Volume**
YOLO^{P}	0.66	**0.76**	**0.8**	0.84	33.32	7.4	**15.13**	104.1
YOLO^{PB}	0.24	0.66	0.7	0.55	34.69	8.33	16.96	105.97
FasterRCNN^{P}	0.3	0.63	0.67	0.71	32.63	9.82	16.31	102
FasterRCNN^{PB}	0.33	0.38	0.66	0.67	33.21	10.03	16.61	95.74
RetinaNet^{P}	0.7	0.6	0.63	0.79	34.42	8.14	17.53	104.87
RetinaNet^{PB}	**0.75**	0.68	0.76	**0.84**	**30.74**	**6.58**	15.88	**89.4**
Mean	0.5	0.62	0.7	0.74	NA	NA	NA	NA

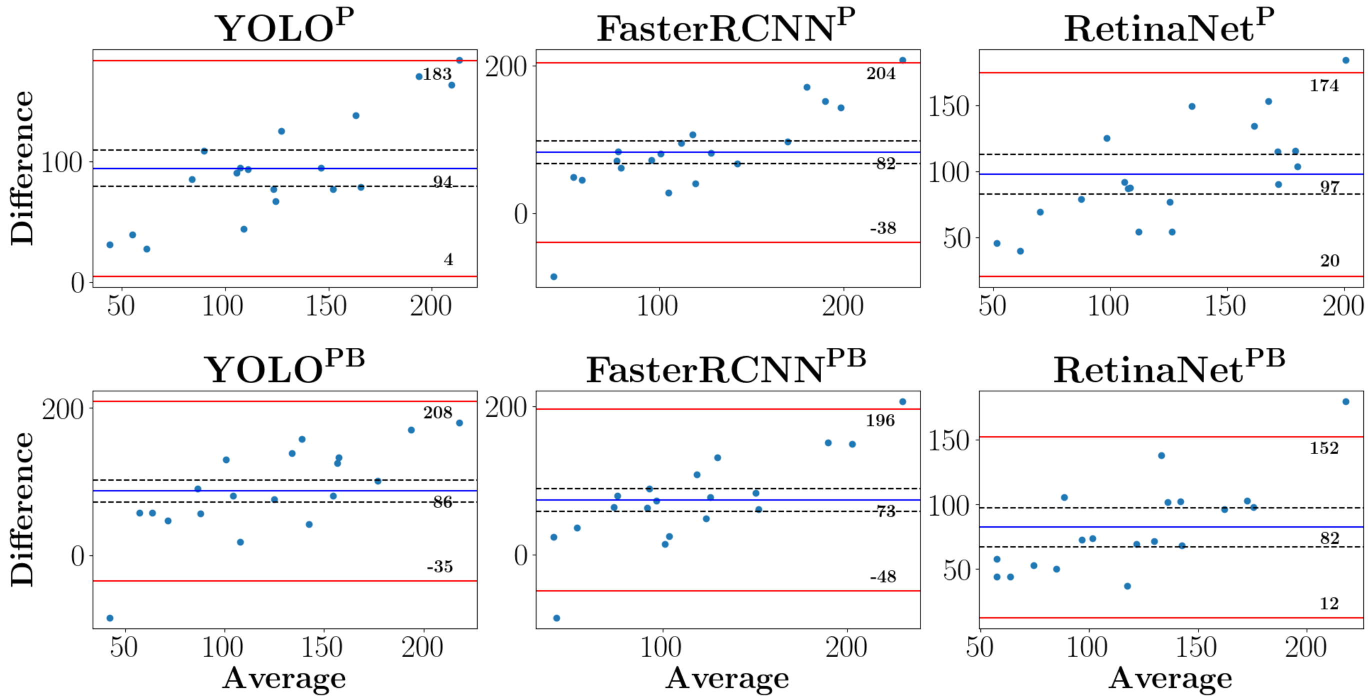

Supplementary Figure 1: 5-fold cross-validation Bland-Altman (difference) volume plots for all models, all units in cm^3. Red lines indicate limits-of-agreement, blue line the mean difference, and the black dashed lines arbitrarily chosen acceptable difference limits (mean ± 15cm^3). Black text indicates numerical values for the limits-of-agreement and mean difference.

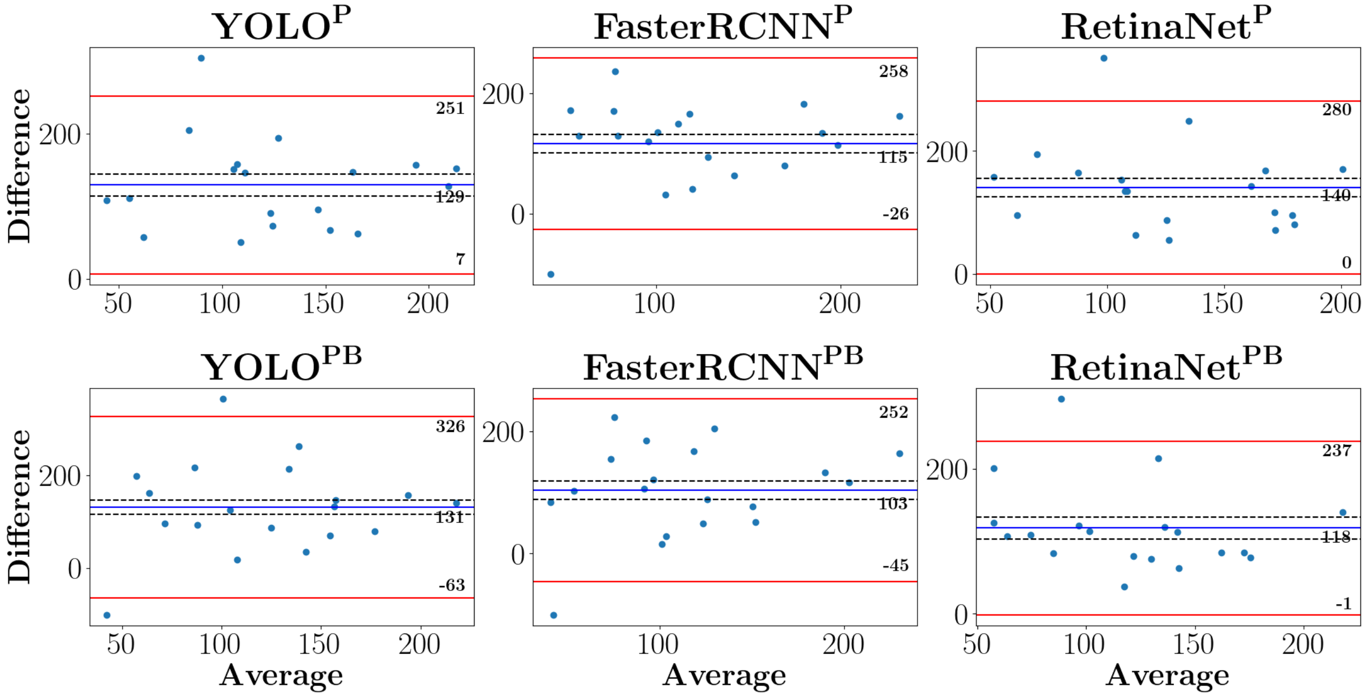

Supplementary Figure 2: 5-fold cross-validation Bland-Altman volume plots for all models, where differences are calculated as a percentage of the true value and the average is in cm^3*. Red lines indicate limits-of-agreement, blue line the mean difference, and the black dashed lines arbitrarily chosen acceptable difference limits (*$\text{mean} \pm 15\%$*). Black text indicates numerical values for the limits-of-agreement and mean difference.*

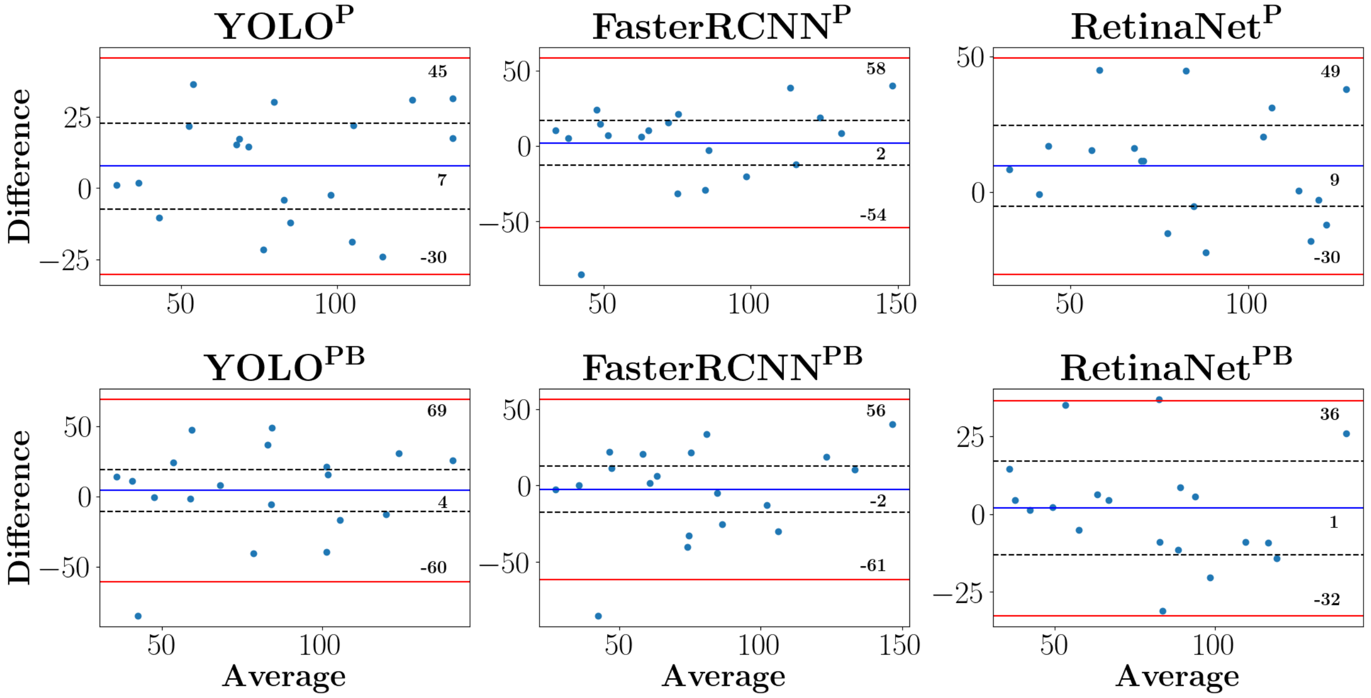

Supplementary Figure 3: 5-fold cross-validation Bland-Altman volume plots for all models, all units in cm^3*. In the volume calculation the SI dimensions was halved to address the overestimation of the SI dimension (as seen by its larger RMSE values in comparison to RL and AP dimensions in the cross-validation study). Red lines indicate limits-of-agreement, blue line the mean difference, and the black dashed lines arbitrarily chosen acceptable difference limits (*$\text{mean} \pm 15\mathrm{cm}^3$*). Black text indicates numerical values for the limits-of-agreement and mean difference.*

Supplementary Table 5: Retrospective metric numeric results for the prospective models. All metrics were calculated for all detected prostate bounding boxes except IoU. Bold values indicate best performing model per metric.*

Model	IoU*	IoU	Precision	F1	Recall	AP50	AP75	AP50-95
YOLO^{P}	0.5	0.48	0.6	0.6	0.6	0.58	**0.32**	**0.33**
YOLO^{PB}	0.48	0.48	0.65	0.65	0.65	0.64	0.27	0.32
FasterRCNN^{P}	0.52	0.42	0.59	0.59	0.59	0.52	0.12	0.21
FasterRCNN^{PB}	0.55	0.47	0.64	0.64	0.64	0.57	0.16	0.25
RetinaNet^{P}	0.54	0.53	**0.76**	**0.76**	**0.76**	**0.74**	0.12	0.29
RetinaNet^{PB}	**0.62**	**0.55**	0.69	0.69	0.69	0.63	0.27	0.32

Supplementary Table 6: Retrospective metric numeric results for the IPV models. All metrics were calculated for all detected prostate bounding boxes except IoU. Bold values indicate best performing model per metric.*

Model	IoU*	IoU	Precision	F1	Recall	AP50	AP75	AP50-95
YOLO^{P}	0.64	0.64	0.84	0.84	0.84	0.84	0.41	0.47
YOLO^{PB}	**0.71**	**0.7**	**0.92**	**0.92**	**0.92**	**0.91**	**0.52**	**0.52**
FasterRCNN^{P}	0.7	0.6	0.77	0.77	0.77	0.71	0.29	0.36
FasterRCNN^{PB}	0.67	0.58	0.76	0.76	0.76	0.7	0.33	0.37
RetinaNet^{P}	0.54	0.53	0.76	0.76	0.76	0.74	0.12	0.29
RetinaNet^{PB}	0.62	0.55	0.69	0.69	0.69	0.63	0.27	0.32

Supplementary Table 7: Retrospective PCC and RMSE numeric results for the prospective models. Only the highest confidence bounding box was used for the inferred dimension calculations. SI, RL, and AP RMSE values are in mm while the Volume RMSE value is in cm^3. Bold values indicate best performing model per measurement.

	PCC				RMSE			
**Model**	**SI**	**RL**	**AP**	**Volume**	**SI**	**RL**	**AP**	**Volume**
YOLO^{P}	0.66	0.45	0.41	0.68	31.62	16.84	**21.35**	**50.94**
YOLO^{PB}	0.39	0.55	**0.57**	0.67	35.5	20.68	22.99	64.55
FasterRCNN^{P}	0.24	0.45	0.22	0.45	29.62	15.11	29.23	90.3
FasterRCNN^{PB}	**0.69**	0.71	-0.06	0.76	**24**	9.48	26.7	71.7
RetinaNet^{P}	0.54	0.62	0.32	0.78	31.34	12.8	24.54	83.81
RetinaNet^{PB}	0.54	**0.73**	0.36	**0.83**	25.77	**9.09**	21.85	65.82
Mean	0.51	0.59	0.3	0.69	NA	NA	NA	NA

Supplementary Table 8: Retrospective PCC and RMSE numeric results for the IPV models. Only the highest confidence bounding box was used for the inferred dimension calculations. SI, RL, and AP RMSE values are in mm while the Volume RMSE value is in cm^3. Bold values indicate best performing model per measurement.

	PCC				RMSE			
**Model**	**SI**	**RL**	**AP**	**Volume**	**SI**	**RL**	**AP**	**Volume**
YOLO^{P}	0.51	0.42	0.63	**0.89**	29.28	14.61	20.93	58.39
YOLO^{PB}	**0.65**	0.32	0.58	0.88	23.67	13.37	**20.17**	**52.48**
FasterRCNN^{P}	0.58	0.51	**0.65**	0.85	22.45	**8.94**	23.08	58.4
FasterRCNN^{PB}	0.62	0.48	0.6	0.83	**21.98**	9.94	22.58	63.23
RetinaNet^{P}	0.54	0.62	0.32	0.78	31.34	12.8	24.54	83.81
RetinaNet^{PB}	0.54	**0.73**	0.36	0.83	25.77	9.09	21.85	65.82
Mean	0.57	0.52	0.52	0.84	NA	NA	NA	NA

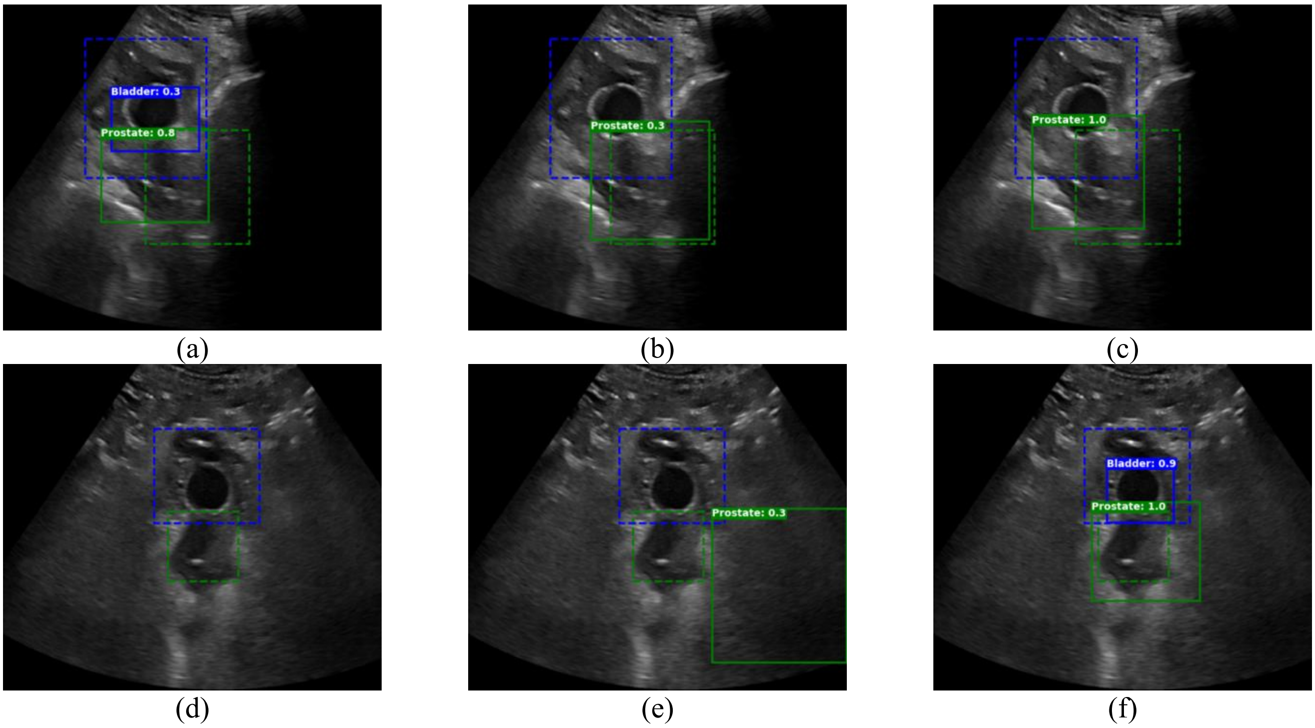

Supplementary Figure 4: Sample special case retrospective results on a patient that had a catheter inserted into their bladder. Left column – YOLO^{PB} results; Middle column – RetinaNet^{PB} results; Right column – FasterRCNN^{PB} results; Top row – Sagittal images; Bottom row – Transverse images Model inferred boxes are solid lines, while ground truth boxes are dashed. Only the highest confidence boxes are shown, with green for prostate and blue for bladder. White text indicates box confidence.

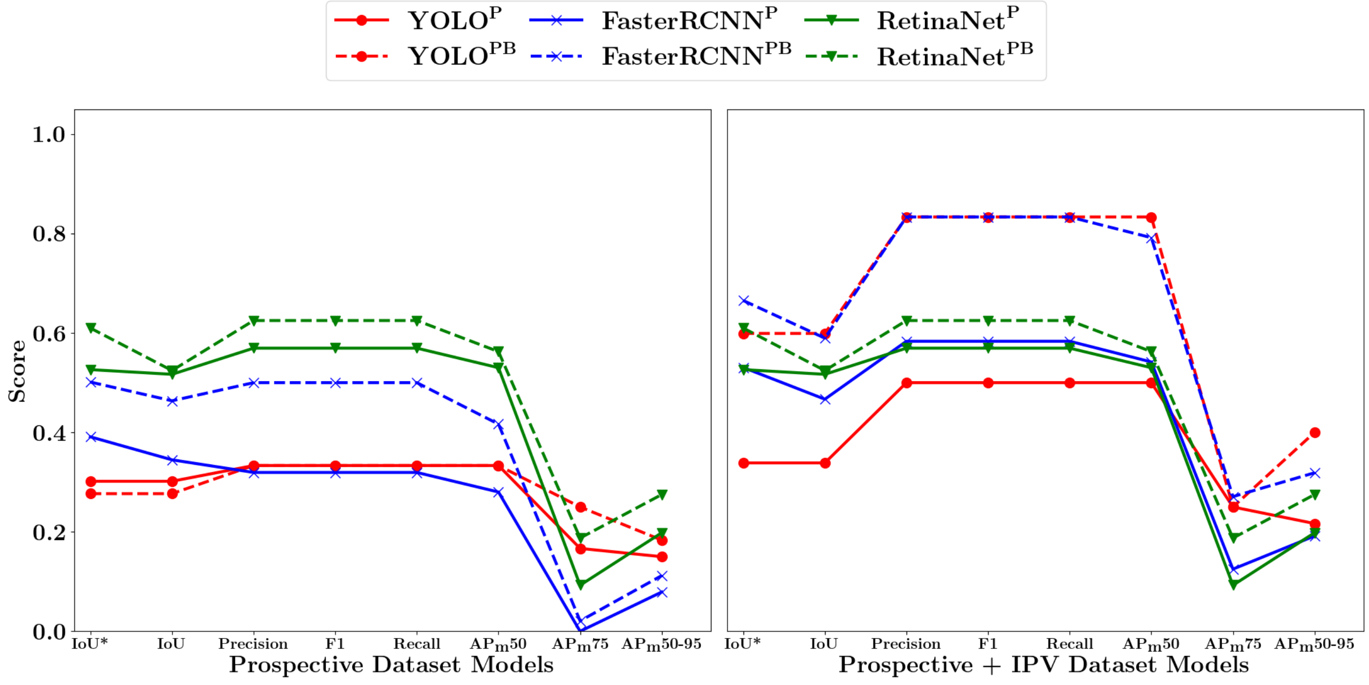

Supplementary Figure 5: Summary of prospective (left) and IPV (right) model metrics on "special case" retrospective images. The dashed lines correspond to models trained to detect both bladder and prostate, with solid lines corresponding to models that were trained to detect prostate only

Supplementary Table 9: Retrospective special case metric numeric results for the prospective models. All metrics were calculated for all detected prostate bounding boxes except IoU. Bold values indicate best performing model per metric*

Model	IoU*	IoU	Precision	F1	Recall	AP50	AP75	AP50-95
$YOLO^{P}$	0.3	0.3	0.33	0.33	0.33	0.33	0.17	0.15
$YOLO^{PB}$	0.28	0.28	0.33	0.33	0.33	0.33	**0.25**	0.18
$FasterRCNN^{P}$	0.39	0.34	0.32	0.32	0.32	0.28	0	0.08
$FasterRCNN^{PB}$	0.5	0.46	0.5	0.5	0.5	0.42	0.02	0.11
$RetinaNet^{P}$	0.53	**0.52**	0.57	0.57	0.57	0.53	0.07	0.2
$RetinaNet^{PB}$	**0.61**	0.52	**0.63**	**0.63**	**0.63**	**0.56**	0.19	**0.27**

Supplementary Table 10: Retrospective special case metric numeric results for the IPV models. All metrics were calculated for all detected prostate bounding boxes except IoU. Bold values indicate best performing model per metric*

Model	IoU*	IoU	Precision	F1	Recall	AP50	AP75	AP50-95
$YOLO^{P}$	0.34	0.34	0.5	0.5	0.5	0.5	0.25	0.22
$YOLO^{PB}$	0.6	0.6	0.8	0.8	0.8	**0.8**	0.25	**0.4**
$FasterRCNN^{P}$	0.53	0.47	0.58	0.58	0.58	0.54	0.13	0.19
$FasterRCNN^{PB}$	**0.67**	**0.59**	**0.83**	**0.83**	**0.83**	0.79	**0.27**	0.32
$RetinaNet^{P}$	0.53	0.52	0.57	0.57	0.57	0.53	0.09	0.2
$RetinaNet^{PB}$	0.61	0.52	0.63	0.63	0.63	0.56	0.19	0.27

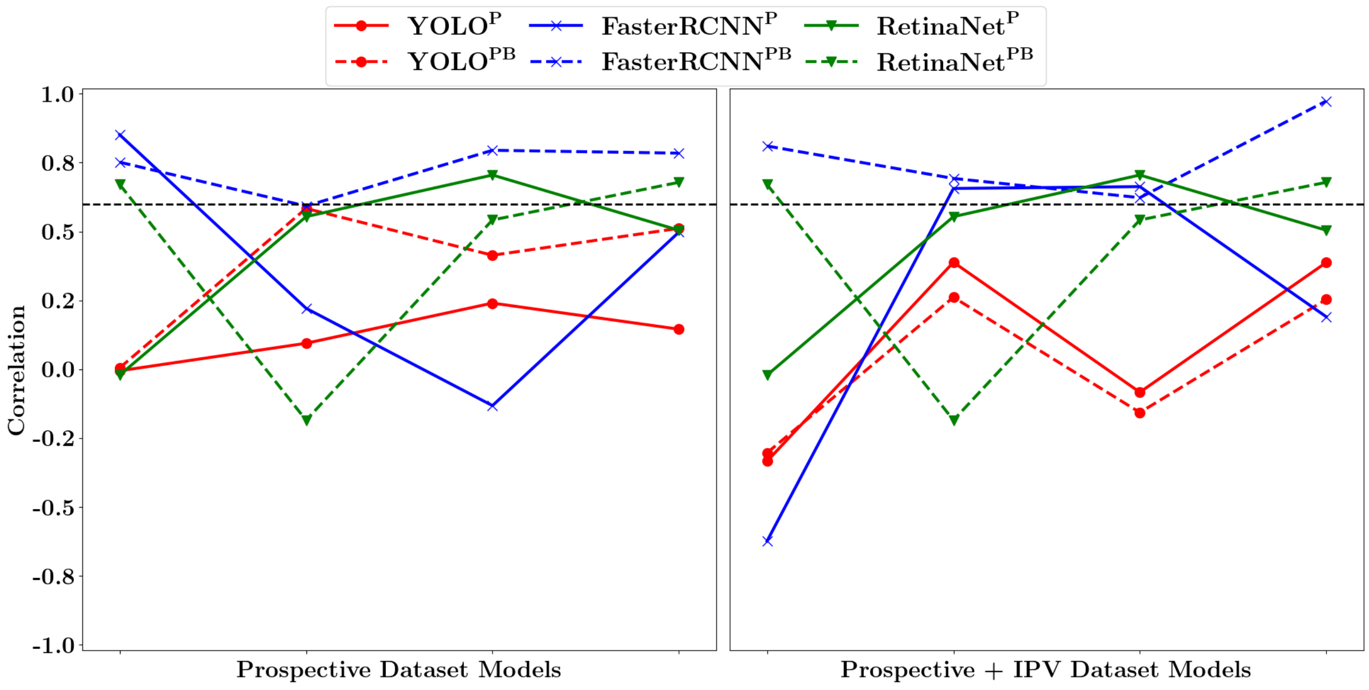

Supplementary Figure 6: Summary of prospective (left) and IPV (right) model PCCs comparing ground truth dimensions with bounding box inferred dimensions for the YOLO (red), RetinaNet (green), and FasterRCNN (blue) models on special case retrospective images only. The dashed lines correspond to models trained to detect both bladder and prostate, with solid lines corresponding to models that were trained to detect prostate only. Only the highest confidence bounding box was used for the inferred dimension calculations. The black lines indicate a PCC of 0.6.

Supplementary Table 11: Retrospective special case PCC numeric results for the prospective and IPV models. Only the highest confidence bounding box was used for the inferred dimension calculations. Bold values indicate best performing model per measurement.

	**Prospective PCC**				**IPV PCC**			
**Model**	**SI**	**RL**	**AP**	**Volume**	**SI**	**RL**	**AP**	**Volume**
$YOLO^{P}$	0	0.09	0.24	0.15	-0.33	0.39	-0.08	0.39
$YOLO^{PB}$	0.01	0.58	0.41	0.51	-0.3	0.26	-0.16	0.25
$FasterRCNN^{P}$	**0.85**	0.22	-0.13	0.5	-0.63	0.66	0.66	0.19
$FasterRCNN^{PB}$	0.75	**0.59**	**0.79**	**0.78**	**0.81**	**0.69**	0.62	**0.97**
$RetinaNet^{P}$	-0.02	0.55	0.71	0.5	-0.02	0.55	**0.71**	0.5
$RetinaNet^{PB}$	0.67	-0.18	0.54	0.68	0.67	-0.18	0.54	0.68
Mean	0.38	0.31	0.43	0.52	0.03	0.26	-0.16	0.25
